# Frequency of speech disruptions in Parkinson's Disease and developmental stuttering: A comparison among speech tasks

**DOI:** 10.1371/journal.pone.0199054

**Published:** 2018-06-18

**Authors:** Fabiola Staróbole Juste, Fernanda Chiarion Sassi, Julia Biancalana Costa, Claudia Regina Furquim de Andrade

**Affiliations:** Department of Speech-Language and Hearing Science, School of Medicine, University of São Paulo, São Paulo, Brazil; University of Pennsylvania Perelman School of Medicine, UNITED STATES

## Abstract

**Objective:**

To analyze the frequency of speech disruptions across different speech tasks, comparing the performance of individuals with Parkinson’s Disease (PD) and DS.

**Method:**

Participants were 20 people with PD, 20 people with DS and 40 fluent individuals. Speech samples were recorded during monologue speech, choral and solo oral reading. Transcriptions of 200 fluent syllables were performed to identify stuttering-like disfluencies (SLDs) and other disfluencies (ODs).

**Results:**

People with PD presented significantly less speech disruptions when compared to people with DS, but significantly more speech disruptions than the control group. Stuttering-like disfluencies ocurred more frequently during monologue speech and solo oral reading for both PD and DS, whereas the control group did not present difference between these tasks.

**Conclusion:**

The stuttering pattern presented by people with PD is different from what is usually described as being neurogenic stuttering.

## Introduction

Parkinson’s disease (PD) is a chronic disease characterized by the progressive degeneration of nigrostriatal dopaminergic neurons responsible for the core motor deficits, and by multifocal involvement of the nervous system leading to additional moto and non-motor systems [1,2]. It is defined by the cardinal symptoms of bradykinesia, rigidity, and tremor. According to the available epidemiological data, PD affects approximately 50 in 100,000 people over the age of 50 years [3,4].

Up to 90% of patients with PD develop speech impairment in the course of their illness [3,5]. The movement impairment deficits observed in these patients often lead to a hypokinetic dysarthria. It is generally accepted that parkinsonian dysarthria emerges due to neurogenic impairments at the respiratory, phonatory and articulatory level [6]. The typical pattern of hypokinetic dysarthria is characterized by speech motor hypophonia, monopitch, monoloudness, short rushes of speech, imprecise articulation, an accelerating speech rate and a greater proportion of inappropriate silent intervals in connected speech [2,3,5,7,8]. Besides all of these speech deficits, PD often include disruptions in fluency [7,9,10]. For this reason, the study of PD speech can offer an opportunity to examine acquired disfluencies of known origin [7,8,10]. The speech disruptions observed in patients with PD are often characterized as neurogenic stuttering, since they are derived from an acquired brain disorder and are not the result of a developmental process [5,11].

The literature typically defines acquired neurogenic stuttering (ANS) as stuttering with an onset that occurs after speech development and is a results of demonstrable neurological damage [12–15]. However, underlying mechanisms of disfluent speech in this disorder remain largely speculative mainly because ANS has been associated with damage to all lobes and hemispheres, with the exception of the occipital lobe, and to other structures of the central nervous system such as the corpus callosum, basal ganglia, brainstem and cerebellum [15–18]. Furthermore, ANS has also been described in diseases of different neurologic etiologies such as stroke, traumatic brain injuries, dementia etc. [19,20]. Stuttering associated with acquired neurologic disorders is characterized by notable involuntary repetitions, prolongation or blocking on sounds or syllables in a manner that interrupts the normal rhythm and flow of speech [3].

Several reports implicate the basal ganglia in fluency behavior [5,7,21,22]. Abnormalities in basal ganglia anatomy, function and connectivity have been reported in developmental stuttering and is the core impaired brain structure in PD [5,22–24]. It has been suggested that the presence of stuttering-like behaviors in PD could be linked to dopamine levels in the basal ganglia and levodopa treatment [4,10]. Studies that report the effects of levodopa on speech fluency are controversial, while some studies suggest that levodopa improves speech fluency [4,25], others suggest that dysfluencies are exacerbated by levodopa [26,27]. Unlike the fluency disturbance acquired by patients with PD, developmental stuttering has an early onset, typically between 2 and 7 years of age [5,7,28–30]. Although the complete pathophysiological mechanism underlying developmental stuttering is still unknown and its cause attributed to multiple factors, the key role of disturbed basal ganglia function has been discussed [2,22,31,32].

The literature describes ANS as having a lot of similarity to developmental stuttering. However, a number of authors have suggested several features that may differentiate ANS from developmental stuttering [11,13,25,33,34]. One of the main differences refers to fluency-enhancing conditions such as singing, choral (or unison) speech, choral (or individual) reading, whispering, altered/delayed auditory feedback and adaptation effect (e.g. successive reading of the same text) [13,14,17,34–36]. While in developmental stuttering these fluency-enhancing conditions bring in a quick reduction in dysfluencies, in ANS this reduction is not observed [34,37]. More recently neuroimaging studies were able to map not only differences in the brain activation of individuals with DS during different speech tasks, but also differences between the brain activation of individuals with DS and normally fluent individuals [38,39]. Overall, monologue tasks and oral reading presented positive correlations between the frequency of speech disruptions (i.e. SLDs) and the activation of the supplementary motor area, precentral gyrus, superior temporal gyrus and basal ganglia and negative correlations between the frequency of SLDs and the activation of the lateral premotor cortex and cerebellum. When compared to normally fluent individuals, patients with DS presented lower activation of the caudate, globus pallidus and putamen of the basal ganglia even during choral speech. Although these speech-task related differences have not been reported in neurogenic stuttering [11–14,22,34–36].

From the brief review above, it seems that individuals with PD would present different speech performance during fluency-enhancing conditions when compared to individuals with developmental stuttering because PD results from a neurologic impairment. However, as the literature reports a positive effect of some fluency-enhancing conditions on PD similar to that observed in DS and both disorders include deficits (acquired and functional respectively) in the basal ganglia, the goal of this study was to analyze the frequency of speech disruptions across different speech tasks, comparing the performance of individuals with PD and DS. Our hypothesis is that individuals with PD will present a frequency of speech disruptions across the different speech tasks similar do individuals with DS and different from what is typically described in the literature for ANS (i.e. no observed fluency-enhancing effect).

## Materials and methods

We conducted cross-sectional study of patients with PD and developmental stuttering. This study received prior approval of the Institution’s Ethics Committee (*Comissão para Análise de Projetos de Pesquisa do Hospital das Clínicas da Faculdade de Medicina da Universidade de São Paulo*—CAPPesq HCFMUSP 940.566) and informed written consent was obtained from all of the participants.

### Participants

Participants for this study were 40 adults who were native speakers of Brazilian Portuguese and who were assigned to two groups. The first group was composed of 20 adults with PD (16 males, 4 females; mean age 63.45 years, standard deviation 8.45). Participants were recruited from the Division of Oral Motor Disorders at *Hospital das Clínicas* of the School of Medicine, University of São Paulo, Brazil. In order to be included in this group, people with PD had to present the following characteristics: diagnosed with idiopathic PD by a neurologist trained in the diagnosis of movement disorders; at least 10 years of formal schooling; score on the Mini-Mental State Exam [40] within normal limits according to exam criteria (i.e. years of formal schooling); no history of previous surgical procedures (i.e. deep brain stimulation–DBS, or stereotaxic surgery); making daily use of anti-Parkinsonian medication (i.e. Levedopa) and considered to be in the on medication state at the time of the speech assessment; free of other neurological diseases; no history of stuttering prior to the diagnosis of PD. According to the Hoehn and Yahr Scale [41], participants included in the PD group were classified as follows: 5 participants as mildly affected (Hoehn-Yahr stage 1.5); 6 participants as moderately affected (Hoehn-Yahr stage 2.5); 9 participants as severely affected (Hoehn-Yahr stages 3 and 4). Patients with PD were making use of anti-Parkinsonian medication for a period that varied between 4 and 15 years.

The second group was composed of 20 adults with developmental stuttering (DS– 17 males, 3 females; mean age 29.55 years, standard deviation 8.92). Participants were recruited from the speech and language clinic at the Department of Speech-Language and Hearing Science of the School of Medicine University of São Paulo, Brazil. In order to be included in this group, patients with DS had to present the following characteristics: diagnosed with idiopathic developmental stuttering by a speech-language pathologist trained in the diagnosis of speech fluency disorders; diagnosed at least with ‘mild’ stuttering as assessed with the Stuttering Severity Instrument– 3 [42]; at least 10 years of formal schooling; free of other speech disorders; free of neurological disorders; no history of prior speech therapy. The stuttering severity of the participants included in the DS group (i.e. according to the SSI-3) was classified as follows: 3 participants with mild stuttering; 8 participants with moderate stuttering; 7 participants with severe stuttering; 2 participants with very severe stuttering.

For comparison purposes, two control groups of 20 fluent healthy adult volunteers each were recruited. In order to be included in this group, individuals had to present the following characteristics: absence of any communication disorders (i.e. language, speech, hearing and oral motor skills) verified by a certified speech-language pathologist; free of neurological or degenerative disorders; no history of prior speech therapy. Individuals in the first control group were matched for age, gender and years of schooling to the participants in the PD group (16 males, 4 females; mean age 63.6 years, standard deviation 8.91) and individuals in the second control group were matched in the same manner to the DS group (17 males, 3 females; mean age 25.6 years, standard deviation 5.87).

### Speech material

For speech fluency evaluation, speech samples of each participant were recorded using an Ipad mini (Apple® 128Gb), in three different conditions—i.e. monologue speech, choral reading with a second speaker, solo oral reading. These speech conditions were selected based on previous research about fluency-enhancing conditions, where monologue speech is considered the baseline condition [12,14]. All recordings were made in a quiet acoustic room at the University of São Paulo.

For the monologue task, individuals either produced a spontaneous monologue on a topic of their choice, or a sample was elicited by prompts given by a therapist. Prior to the monologue, participants were given suggestions as to topics, such as family, sports, friends, hobbies, films, actual television. Individuals were asked to speak continuously on these topics, and the topics were suitable for all ages. If participants were not able to speak continuously, prompting questions were made by the examiner to encourage speech. The length of the recordings for the groups was similar; an average of 3 minutes of speaking time (i.e. time necessary to obtain 200 fluent syllables). A sample of 200 fluent syllables was selected for analysis for each individual (i.e we considered the first 200 fluent syllables). For the oral reading task, the reading was performed solo and the participants read a passage containing 200 syllables. For the choral reading task, the participants simultaneously read a passage containing 200 syllables with the clinician. We used different passages for the oral and choral reading tasks to avoid the adaptation effect. Both passages were taken from a popular magazine.

#### Speech sample analysis

The speech samples from all participants were randomly assigned to two certified speech-language pathologists, trained in the field of stuttering. They had no knowledge to which group each participant belonged. Orthographic transcriptions were carried out and the stuttering episodes within the 200 syllables were marked on the transcripts. For the monologue task, single word answers such as “yes” or “no” in response to prompting questions were excluded from analysis. For each participant the frequency of stuttering-like disfluencies (SLD) and other disfluencies (OD) was calculated [43]. SLD included one-syllable word repetitions, sound and syllable repetitions, sound prolongations, and blocks. Multi-syllable word repetitions, phrase repetitions, interjections, revisions, and interrupted utterances were defined as OD.

#### Reliability measures

Speech fluency assessment (identification of the stuttering-like disfluencies and other disfluencies) was performed by the primary investigator. The same investigator re-evaluated speech fluency for 10% of the speech samples, chosen at random (a total of 30 speech samples) 6 months after initial analysis. Intrajudge agreement was 0.92, as indexed by Cohen's kappa coefficient. Kappa values above 0.75 represent excellent agreement beyond chance. A second judge, a trained speech language pathologist, also independently assessed speech fluency for 10% of the speech samples chosen at random. Interjudge agreement was 0.85 as indexed by Cohen's kappa coefficient.

### Data analysis

The distribution of the data was non-normal for all variables. For this reason, the analysis was performed using non-parametric tests. In addition to the descriptive analysis, multiple comparisons among the groups were performed using the Kruskal-Wallis test and the Friedman ANOVA for within group comparisons. The Dunn’s test was used for the post hoc pairwise analysis. The adopted significance level was of 5% for all analysis.

Prior to performing the comparisons among the groups, we performed a between-group analysis only for the control groups. Even though these groups presented a significant difference in age (p = 0.0001*) according to the Kruskal-Wallis test, they did not differ in fluency performance (p = 0.588 for SLDs and p = 0.448 for ODs during the monologue speech; p = 0.665 for SLDs and p = 0.715 for ODs during solo reading; p>0.999 for SLDs and p>0.999 for ODs during choral reading). For this reason all the control individuals were considered a single group for the statistical analysis.

## Results

Overall, 240 speech samples were available for observation and analysis. As specified in the method section, only the first 200 fluent syllables (and the speech disruptions within these 200 syllables) were analyzed for all of the participants.

**Fig 1:**
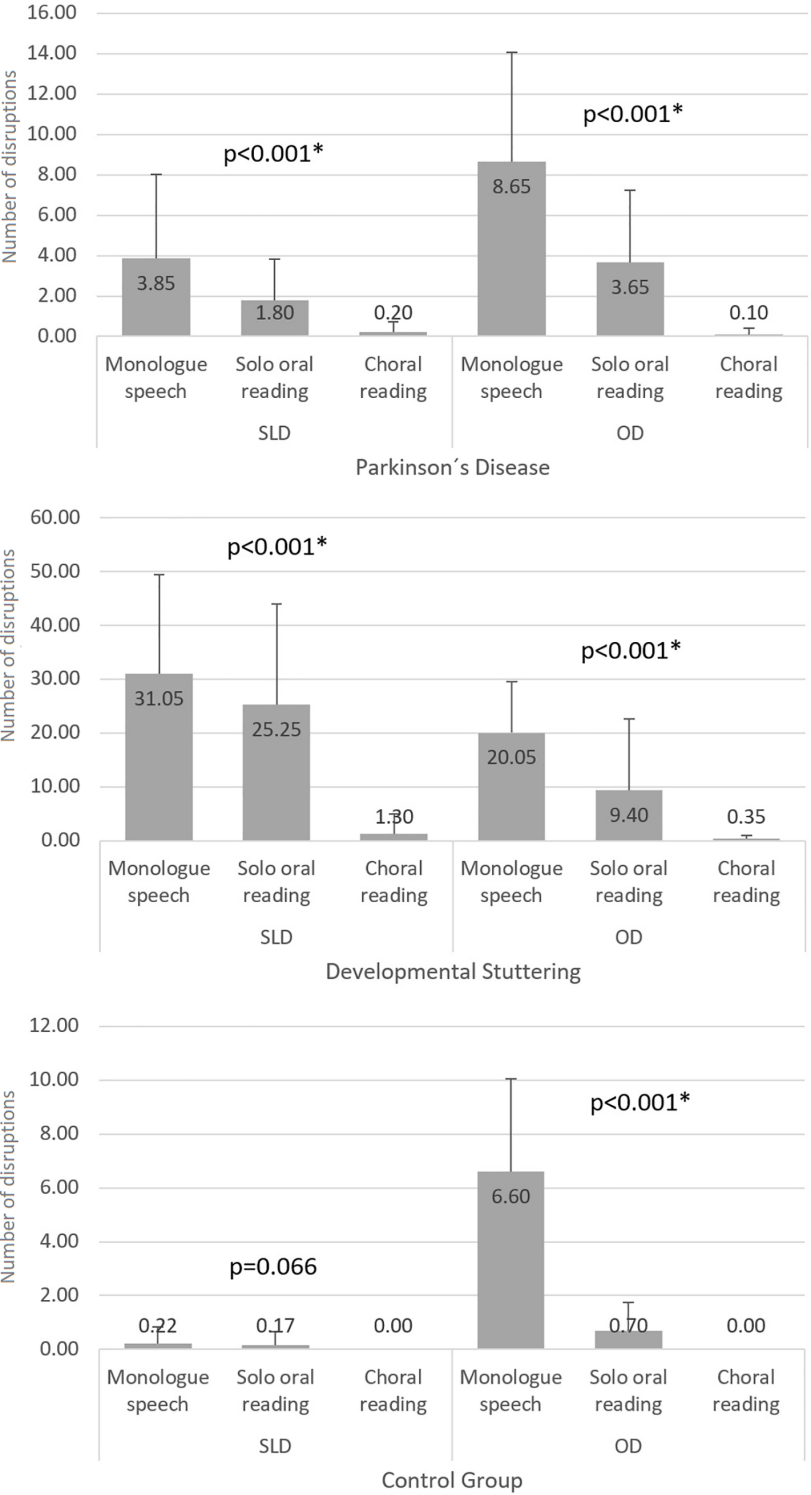
Within group comparison for the different speech tasks. SLD–stuttering-like disfluencies; OD–other disfluencies; * significant results; Friedman ANOVA test.

Fig 1 shows the results of the within group comparison for the different speech tasks.

Results indicated that patients with PD presented more SLDs during the monologue speech task and during the solo oral reading (monologue speech vs solo oral reading—p = 0.537; monologue speech vs choral reading–p<0.001; solo oral reading vs choral reading–p = 0.017, according to the Dunn-Bonferroni post hoc method). The same performance was observed for patients with DS–i.e. SLDs occurred more frequently during the monologue speech task and during solo oral reading (monologue speech vs solo oral reading–p>0.999; monologue speech vs choral reading–p<0.001; solo oral reading vs choral reading–p<0.001, according to the Dunn-Bonferroni post hoc method). SLDs did not vary among the different speech tasks for the control group.

Regarding the frequency of ODs among the speech tasks, the groups presented a similar behavior to that observed for the frequency of SLD: patients with PD presented more ODs during the monologue speech task and during the solo oral reading (monologue speech vs solo oral reading—p = 0.098; monologue speech vs choral reading–p<0.001; solo oral reading vs choral reading–p = 0.003, according to the Dunn-Bonferroni post hoc method); patients with DS also presented more ODS during the monologue speech task and during the solo oral reading (monologue speech vs solo oral reading—p = 0.098; monologue speech vs choral reading–p<0.001; solo oral reading vs choral reading–p = 0.003, according to the Dunn-Bonferroni post hoc method); the control group did not present ODs during choral reading and presented different performances during the monologue speech and the solo oral reading (monologue speech vs solo oral reading–p<0.001; monologue speech vs choral reading–p<0.001; solo oral reading vs choral reading–p = 0.221, according to the Dunn-Bonferroni post hoc method).

Tables 1 and 2 show the results of the between-group analysis for the frequency of SLDs and ODs respectively.

**Table 1:** Stuttering-like disfluencies–between-group comparisons.

Speech task	Group	Mean(±SD)	H	df	p-value	Pairwise analysis
Monologue speech	PD	3.85(±4.18)	65.76	2	<0.001*	PD≠DS p = 0.005* PD≠CG p<0.001* DS≠CG p<0.001*
	DS	31.05(±18.37)				
	CG	0.22(±0.61)				
Solo oral reading	PD	1.80(±2.01)	59.49	2	<0.001*	PD≠DS p<0.001* PD≠CG p = 0.008* DS≠CG p<0.001*
	DS	25.25(±18.73)				
	CG	0.17(±0.50)				
Choral reading	PD	0.20(±0.52)	12.49	2	0.002*	PD = DS p = 0.337 PD = CG p = 0.290 DS≠CG p<0.001*
	DS	1.30(±3.61)				
	CG	0(±0)				

PD–Parkinson’s disease; DS–developmental stuttering; CG–control group; SD–standard deviation; df—degrees of freedom

* significant results; Kruskal-Wallis test and Dunn-Bonferroni test.

**Table 2:** Other disfluencies–between-group comparisons.

Speech task	Group	Mean(±SD)	H	df	p-value	Pairwise analysis
Monologue speech	PD	8.65(±5.40)	32.58	2	<0.001*	PD≠DS p = 0.002* PD = CG p = 0.212 DS≠CG p<0.001*
	DS	20.05(±9.43)				
	CG	6.60(±3.46)				
Solo oral reading	PD	3.65(±3.58)	29.14	2	<0.001*	PD = DS p>0.999 PD≠CG p<0.001* DS≠CG p<0.001*
	DS	9.40(±13.22)				
	CG	0.70(±1.04)				
Choral reading	PD	0.10(±0.30)	10.52	2	0.005*	PD = DS p = 0.255 PD = CG p = 0.633 DS≠CG p = 0.004*
	DS	0.35(±0.67)				
	CG	0(±0)				

PD–Parkinson’s disease; DS–developmental stuttering; CG–control group; SD–standard deviation; df–degrees of freedom

* significant results; Kruskal-Wallis test and Dunn-Bonferroni test.

The results indicated that patients with DS present significantly more SLDs during the monologue speech and solo oral reading when compared to the patients with PD and to the control group. Although patients with PD presented fewer SLDs when compared to patients with DS, they still produced significantly more SLDs than the control group in both speech tasks. The speech task that least differentiated the groups was choral reading. The only observable significant difference was between patients with DP and the control group. (i.e patients with DP presented more SLDs during the choral reading than the control group). A different behavior was observed when analyzing the frequency of ODs. Patients with PD presented similar results to patients with DP during the solo oral reading and during the choral reading. In this analyzes, patients with PD were more similar to the control group (i.e. no observable significant difference during the monologue speech and the choral reading). Patients with DS produced more ODs than the control group in all speech tasks.

## Discussion

The aim of the current investigation was to determine if individuals with PD exhibit stuttering-like behaviors similar to individuals with DS and also to determine if these behaviors exhibit significant variations during fluency enhancing conditions. Overall, patients with PD presented significantly less speech disruptions when compared to patients with DS, but significantly more speech disruptions than the control group. Suttering-like disfluencies ocurred more frequently during the monologue speech task and during solo oral reading for both PD and DS, whereas the control group did not present significant difference between these tasks.

Among the studies of speech disruptions in individuals with DS, SLDs are considered to represent the differences in motoric ability between people who stutter and those who are normally fluent [3,22,28,30,44–46]. While normal disfluencies (i.e.ODs) typically occur at the syntactic or semantic level, SLDs are observed at the phonemic level [3,22,30,44]. According to the the theory proposed by Howell [45]—EXPLAN model, language planning and execution are parallel independent processes with neither process being monitored for errors. Speech disruptions ocurrs if an individual speaks fast and finishes executing one segment before the plan for the next segment is ready. Fluency failure is then viewed as a sign that planning and execution processes are out of synchrony. In neurologic terms, the symptoms of stuttering are compared with basal ganglia motor disorders like Parkinson’s disease and dystonia [22]. It is proposed that the basal ganglia-thalamocortical motor circuits through the putamen are likely to play a key role in stuttering. Briefly, the core dysfunction in stuttering is suggested to be impaired ability of the basal ganglia to produce timing cues for the initiation of the next motor segment in speech [22]. In our study participants with PD presented an overall range of SLDs relatively smaller in comparison to participants with DS (i.e patients with DS presented ten times more SLDs during the monologue speech task). According to the literature, the presence of SLDs in patients with PD suggests that they exihibit a motoric-based stuttering, which may be related to the motor-coordination deficits inherent to the disease [5,7,10]. The presence of SLDs in the speech of patients with PD might be explained by difficulties to initiate speech motor commands secondary to basal ganglia dysfunction associated to fluctuations in the dopamine level [5]. Although all of our patients were making daily use of anti-Parkinsonian medication, the literature points that after the initial 5-year period of stable response to L-dopa (i.e. main ingredient in the medication usually given to patients with PD), patients may experience motor side effects [47], such as stuttering [3]. Nevertheless, as the administration of L-dopa produces changes in the levels of dopamine in the brain, it would be possible to speculate that this medication could have an influence on speech production, explaining why the frequency of SLDs in the speech of patients with PD was lower when compared to patients with DS. This point needs further investigation.

We also found differences in the frequency of ODs when comparing patients with PD and DS. When considering this type of speech disruption, patients with PD presented a closer pattern to that observed for the control group. According to the literature, normal disfluencies or ODs may be present in high frequencies in developmental stuttering, but are often observed in the speech of normally fluent individuals [44,48]. The occurrence of ODs is directly related to the linguistic planning of speech [3,22,28,30,44–46]. This category of speech disruptions may reflect linguistic uncertainties and/or imprecisions, and can be used as an additional resource to produce timing cues for the other processes involved in the production of fluent speech [7]. Several researchers have suggested that patients with PD can present some level of language impairment [6,22,49]. This observation is based on models which propose that the basal ganglia and thalamus are involved in language processes and cognition [22,50].

Our study also analyzed differences in performance across three different speech tasks. We found significant speech task differences for both patients with PD and DS. A fluency-enhancing effect has been reported in the literature for patients with acquired neurogenic stuttering secondary to basal ganglia dysfunction like PD [5,18]. According to the authors, it is possible that acquired stuttering secondary to dysfunction of the striatal region may lead to a fluency disorder that is distinct from other forms of acquired neurogenic stuttering. This would explain why patients with PD have speech fluency disturbances that are more similar to patients with DS than to patients with acquired neurogenic stuttering. Our data support the suggestion that the fluency adaptation observed for patients with PD is similar to patients with DS, i.e. both groups presented a significant reduction in SLDs when comparing the monologue task to choral reading. Most patients with DS present a dramatic decrease in stuttering frequency during choral reading, often coming close to fluent levels [14,21,22,34,51]. It has been proposed that during choral reading, the voice of the other person provides external timing cues during speech production, improving speech fluency [22]. Moreover, the literature suggests that the choral speech effect is a form of direct imitation, a primitive human capacity that is mediated by the mirror neurons system [52]. Patients with PD can achieve improved motor ability without external cues, merely by being instructed to consciously attend to a particular aspect of the movement [53]. Individuals with PD were found to demonstrate a significant reduction in the frequency of SLDs when asked to reduce speech rate and to exaggerate speech articulation [7,54]. Both of these therapy techniques have long been reported to have the same effect on the speech of individuals with DS. A possible neurologic explanation for these observations is that structures outside the basal ganglia system have the ability to provide internal timing cues for movement sequences (e.g. speech), but only during non-automatized movements [22].

The present study has a few limitations. First, patients with PD and DS were heterogeneous in terms of severity. It is expected for more severe forms of both disorders to present higher frequencies of speech disruptions, and for this reason our results cannot be generalized. Second, due to its observational character, it was not possible to assess patients with PD in the off-medication state. Therefore, we could not verify the real effects of medication on speech fluency. Nevertheless, the literature has suggested no significant differences in speech fluency in patients with PD in the on versus off state [6,10].

Finally, we would like to consider that our study contributes to the knowledge that the stuttering pattern presented by patients with PD is somewhat different from what is described in the literature as being neurogenic stuttering (i.e. patients with neurogenic stuttering do not present differences across speech tasks and do not present improvement in speech performance during fluency-enhancing conditions). Moreover, the study of speech disruptions in patients with PD may help in the treatment of speech production deficits, as speech disruptions may present a significant challenge for these patients. Our results suggest a possible link between the fluency disorder presented by patients with PD and DS, as both groups seem to benefit from fluency enhancing-conditions. Further work will examine similarities and differences between the outcomes of patients with PD and DS after using traditional motor learning therapeutic strategies currently available for patients with DS.

## Supporting information

S1 Fig Within group comparison for the different speech tasks. (XLSX)
